# Unusual Root Canal Morphology in Mandibular Molars: A Case Series

**DOI:** 10.7759/cureus.115749

**Published:** 2026-09-03

**Authors:** Shelja Mohan, Arjama Chaudhury, George Thomas, Sunil Jose, Sibi Aboobacker

**Affiliations:** 1 Conservative Dentistry and Endodontics, Mahe Institute of Dental Sciences and Hospital, Mahe, IND; 2 Conservative Dentistry and Endodontics, Sri Sai College of Dental Surgery and Hospital, Vikarabad, IND

**Keywords:** c-shaped canal, paramolaris, root canal treatment, rotary endodontics, unusual anatomy

## Abstract

The complex root canal anatomy of mandibular molars often poses a challenge for endodontic treatment. To overcome this, precise access, cleaning, shaping, and three-dimensional obturation are essential. This case report presents three unusual cases of mandibular molars, where rare root canal configurations were accurately diagnosed using cone beam computed tomography (CBCT), and sonic activation was used for disinfection. This report highlights the importance of utilizing 3D imaging and precise canal identification to tackle rare anatomical variations in mandibular molars, which have been rarely documented in the existing literature. This report aims to contribute to understanding and managing the rare and complex root canal configurations in mandibular molars.

## Introduction

Root canal morphology has been extensively studied; however, its variations continue to challenge clinicians and endodontists. Successful endodontic outcomes are associated with a good understanding of root canal anatomy, morphology, and clinical thoroughness, including negotiation, cleaning, shaping, and obturation of the root canal system.

The morphology of root canals has been documented by Vertucci, with emerging variations of canals being updated continuously [1,2]. However, undetected extra roots or unusual root canals, though rare, are encountered during endodontic treatment and are major reasons for the failure of root canal treatment [3,4]. Managing such unexpected variations requires an alert clinician, a proper radiographic technique, and proper interpretation for a sound diagnosis to recognize and manage unusual anatomical configurations, as these can significantly impact treatment outcomes [5].

Cone beam computed tomography (CBCT) is increasingly preferred over conventional radiography. It has been proven to identify extra roots in mandibular first molars and diagnose unusual cases, including those with unusual root canal anatomy [6]. It enables the clinician or endodontist to make better decisions and diagnoses before treatment [7].

## Case presentation

The following case report describes the anatomical variations or aberrations encountered during a routine endodontic procedure [4,8]. The three cases presented in the study are unique. A few similar studies also showed anatomical variations. However, no studies showed the same variations as our case study.

Case 1

A 26-year-old man presented to the Department of Conservative Dentistry and Endodontics with pain in the lower left posterior tooth region. Clinical examination revealed deep distoproximal caries involving tooth #37, which was tender to vertical percussion. Radiographic examination confirmed involvement of the pulp in addition to the presence of an unusual root anatomy in the mesial root. Both clinical and radiographic findings led to a diagnosis of acute apical periodontitis in relation to tooth #37. A CBCT image revealed a bifurcated root originating from the distolingual aspect of the mesial root of tooth #37 that was obliquely displaced towards the mesial aspect of the distal root with partially calcified root canals (Figure 1A).

**Figure 1:**
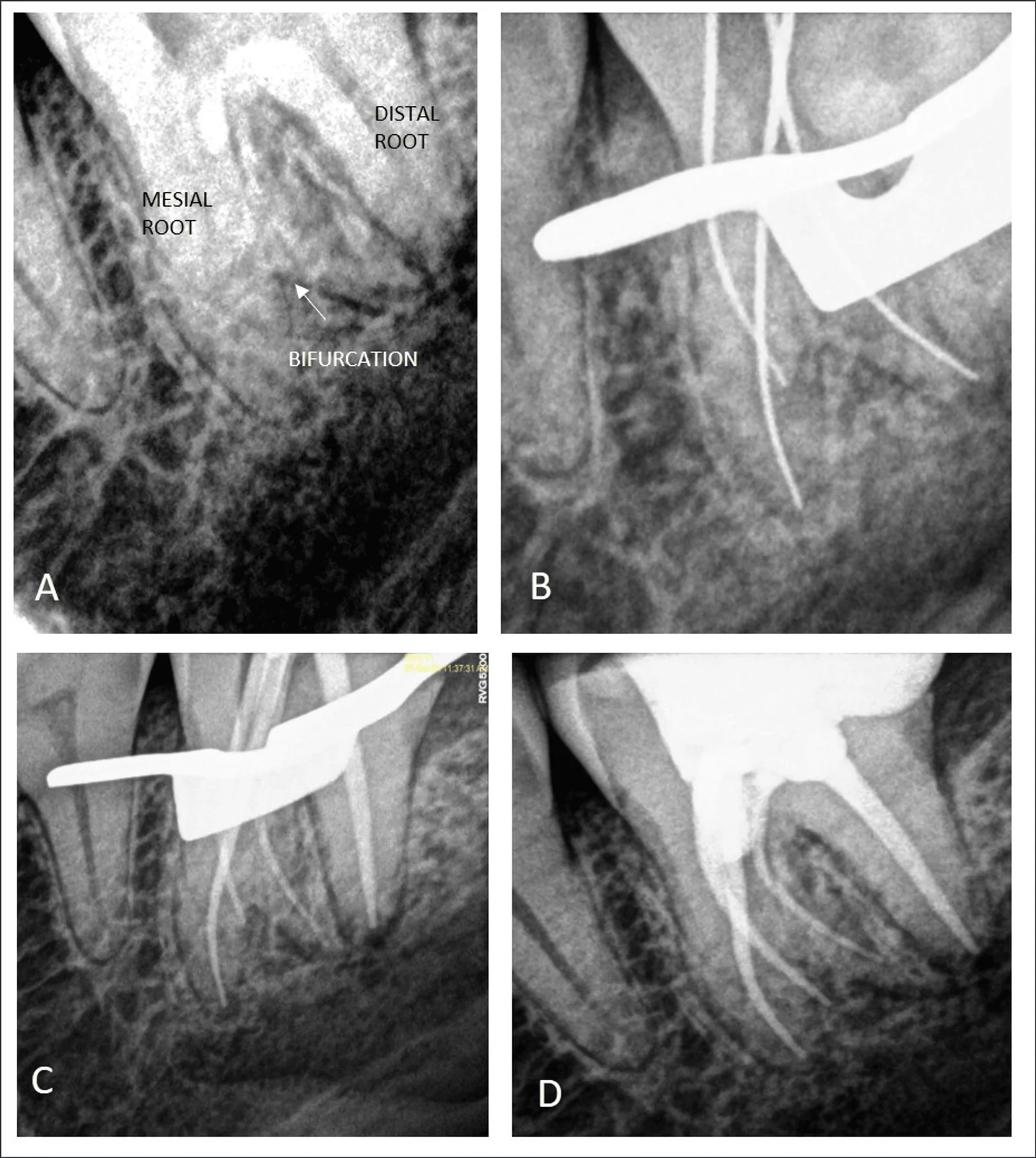
Bifurcated root morphology Figure 1A shows an image of tooth #37 highlighting the bifurcated root originating from the distolingual aspect of the mesial root. Figure 1B shows a working-length radiograph of the root canals in the mesial root. Figure 1C shows a cone-fit radiograph confirming the length and fit of the gutta-percha, while Figure 1D shows a post-obturation radiograph demonstrating complete obturation.

The treatment plan included endodontic management of tooth #37, which was explained to the patient, and informed consent was obtained before the initiation of therapy. The left inferior alveolar nerve was anesthetized using 2% lignocaine (Lignox) with 1:80,000 adrenaline. The tooth was isolated using a rubber dam, and an endodontic access cavity was established. Root canal orifices were located: four in the mesial root and three in the distal root. The canals were explored, and a glide path was established using #6, #8, and #10 K-files (Dentsply Sirona, Charlotte, North Carolina, USA).

Several working-length radiographs were taken at different angulations with files in each canal. An apex locator (Root ZX Mini; J. Morita MFG. CORP., Kyoto, Japan) was used to confirm the working length (Figure 1B). The root canals were cleaned and shaped using a crown-down method with ProTaper Gold instruments (Dentsply Maillefer, Ballaigues, Switzerland) with abundant irrigation of 5.25% sodium hypochlorite (NaOCl), 17% ethylenediaminetetraacetic acid (EDTA), and saline, and activated using an EndoActivator® (Dentsply Sirona). The final rinse was done with normal saline. The root canals were dried using sterile paper points and obturated with a single cone of ProTaper gutta-percha using ProRoot Endo as a sealer (Dentsply Tulsa Dental Specialties, Tulsa, Oklahoma, USA) (Figures 1C-1D).

Case 2

A 32-year-old male patient presented to our department with a chief complaint of pain and discomfort in the lower left back tooth region for the last two weeks. The patient gave a history of pain that was dull and throbbing in nature. On clinical evaluation, a deep dentinal caries was found on the occlusal surface of tooth #37. Radiographic examination revealed a conical-shaped, fused mesial and distal roots with a thin radiolucent line between the roots, suggesting a C-shaped root canal (Figure 2A).

**Figure 2:**
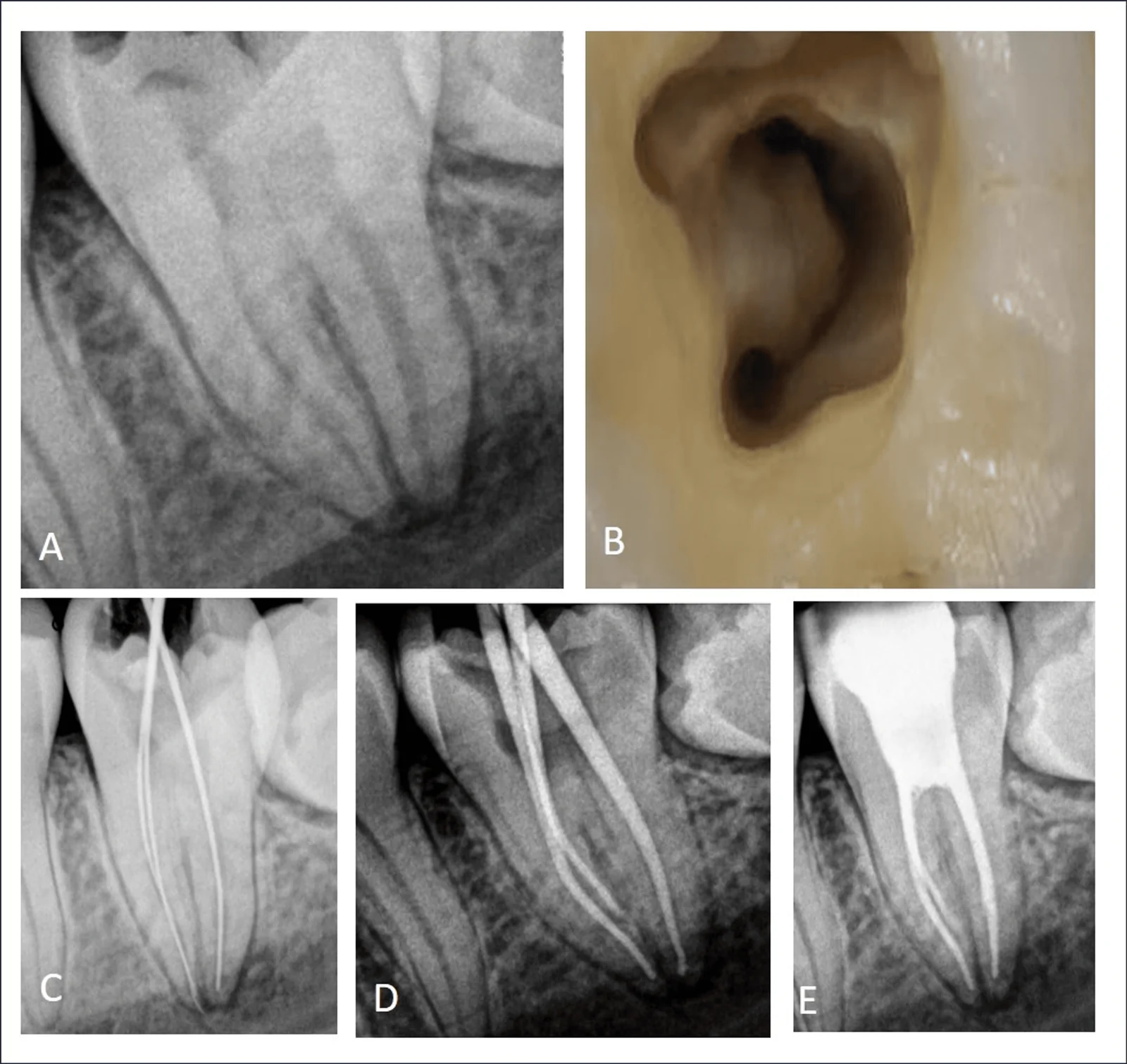
C-shaped canal morphology in a mandibular molar Figure 2A shows a preoperative radiograph of tooth #37. Figure 2B shows the C-shaped canal configuration after access cavity preparation. Figure 2C shows the working-length radiograph. Figure 2D shows a cone-fit radiograph confirming the length and fit of the gutta-percha, while Figure 2E shows a post-obturation radiograph demonstrating complete obturation.

The left inferior alveolar nerve was anesthetized using 2% lignocaine with 1:80,000 adrenaline (Lignox). The tooth was isolated using a rubber dam, and an endodontic access cavity was established. Root canal orifices were located, which revealed a C3-type canal anatomy (Figure 2B) [9]. The working length was determined (Figure 2C), followed by cleaning and shaping the canal using ProTaper Gold nickel-titanium (NiTi) rotary files. The circumferential filing was performed using hand K-files. The canals were then irrigated with 3% NaOCl and activated with EndoActivator®. An intracanal medicament (calcium hydroxide) was placed, and the patient was recalled after one week for follow-up. A follow-up radiograph was taken with the master cone to confirm the working length (Figure 2D). The canals were obturated using gutta-percha, and post-endodontic restoration was done using a composite resin (Figure 2E).

Case 3

A 27-year-old male patient presented with a chief complaint of extreme pain and discomfort in the lower left back tooth region for the past three days. The pain was intermittent, aggravated by consuming hot food and drinks, and lasted for two to three hours. Clinical examination revealed secondary caries on restored tooth #36. Radiographic examination confirmed that the restoration approximated the pulp and revealed the presence of an additional root. The buccal inclination of the distal canal suggested the presence of an additional canal located more lingually (Figure 3A).

**Figure 3:**
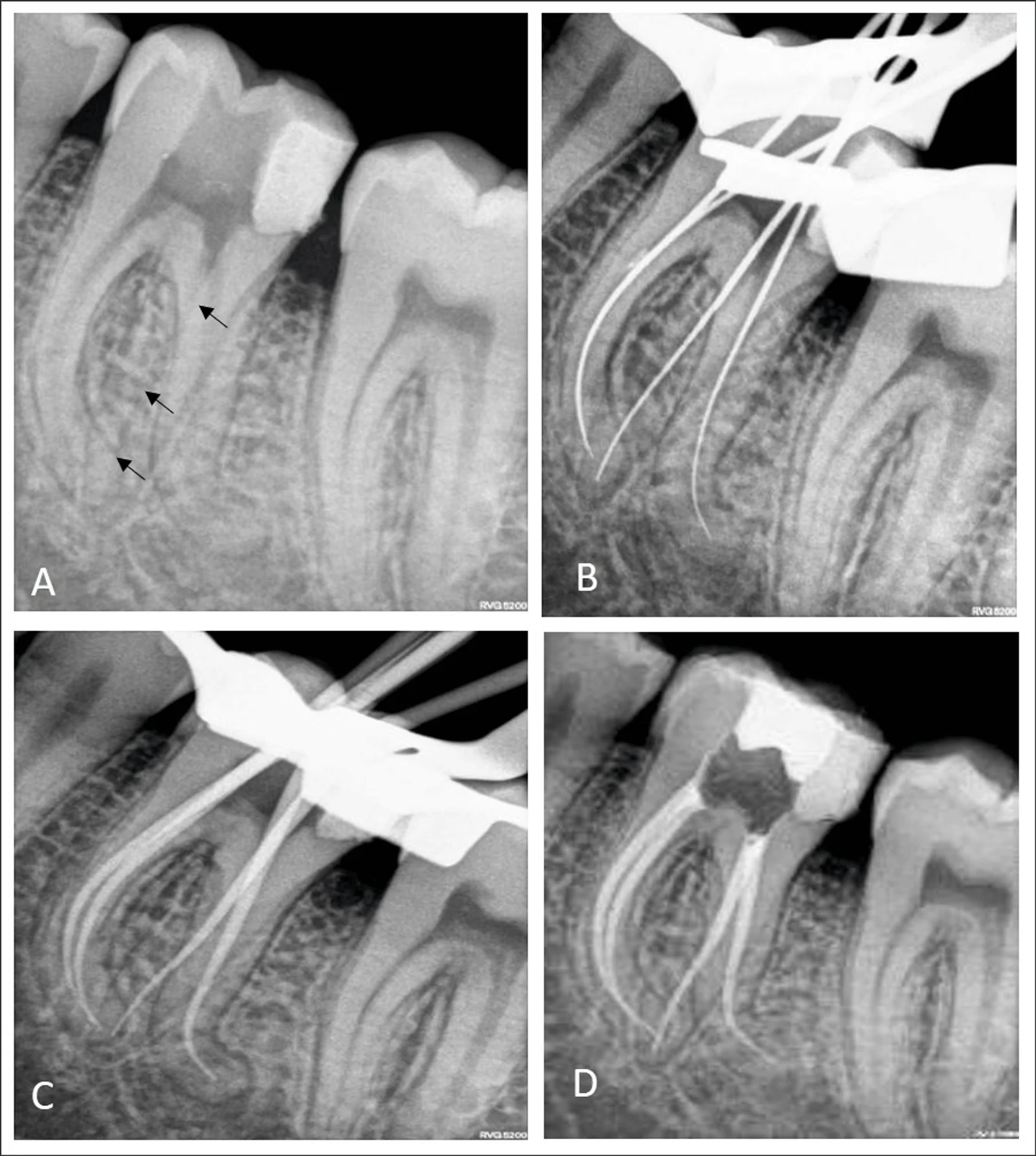
Radix entomolaris: an anatomical variation Figure 3A shows a preoperative radiograph demonstrating the presence of an additional root (arrows). Figure 3B shows the working-length radiograph. Figure 3C shows a cone-fit radiograph confirming the length and fit of the gutta-percha, while Figure 3D shows a post-obturation radiograph demonstrating complete obturation.

The left inferior alveolar nerve was anesthetized using 2% lignocaine with 1:80,000 adrenaline (Lignox). The tooth was isolated using a rubber dam, and an endodontic access cavity was established. The shape of the access cavity was modified from triangular to trapezoidal form to locate the fourth canal. The root canal orifices were located with the help of a DG-16 endodontic explorer (Dentsply Sirona), and a #15 K-file was used to establish patency of the canals. An apex locator was used to determine the working length (Figure 3B). ProTaper Gold rotary files were used for cleaning and shaping the canals with abundant irrigation using NaOCl, and 17% EDTA was used as a final flush. Master cone fit was checked (Figure 3C), followed by obturation with gutta-percha points and ProRoot Endo Sealer. Restoration of the access cavity was done with composite resin cement, and a post-obturation radiograph was taken (Figure 3D).

## Discussion

Understanding and addressing anatomical variations in root canals is critical to the success of endodontic treatments. Root canal systems exhibit a wide range of complexities, such as unusual curvatures, C-shaped canals, extra canals, etc., which can pose significant challenges during treatment and impact the outcome. Clinicians must be aware that the standard root canal morphology outlined in textbooks is not universal. Teeth, particularly molars, often exhibit additional or atypical canals. The failure to detect and treat these canals is a common cause of persistent infection and post-treatment complications.

Digital radiographic imaging coupled with an electronic apex locator can enhance precision in determining the working length, especially in teeth with complex canal anatomy [10]. A careful examination of pre-operative radiographs from multiple angles can assist in identifying the number of canals and their configuration. Furthermore, clinical alertness to subtle tactile changes in the positioning and deformation during instrumentation is vital.

Previous published case reports have documented variations in the root canal anatomy. Neelakantan et al. reported that C-shaped canals were observed in 7.5% of the teeth [11]. Ravi and Honwad reported a C-shaped root morphology with four canals in the mandibular first molar identified using CBCT [12]. Fan et al. reported that types IV and I root canal anatomy were commonly observed among mesial and distal roots of two-rooted second molars [9]. Similarly, Kuzekanani and Najafipour observed that the incidence of radix paramolaris was 1.2% in mandibular first molars and 0.8% in mandibular second molars, respectively [13]. Furthermore, Sheth et al. reported the prevalence of distal lingual root to be 6.62% [14].

Treating teeth with anatomical variations requires a meticulous approach. Conventional hand instrumentation, while effective, may be insufficient in complex canal curvature cases. Procedural errors should be anticipated due to curved and often narrow canals. Such canals require the use of instruments that are heat-treated and resistant to fractures. The introduction of rotary and reciprocating instrumentation systems has improved the ability to navigate complex canal systems. In the present case series, we used C+ files and ProTaper Gold NiTi files for cleaning and shaping.

While addressing the mechanical challenges of anatomical variation, clinicians must not overlook the biological aspects of endodontic treatment. Proper disinfection of the root canal system is essential to eliminate the bacterial load. In line with the protocol, we used 5.25% NaOCl and 17% EDTA as the main irrigants, while EndoActivator® was used for passive irrigation. Such passive irrigation has been noted for its ability to enhance the deep penetration of irrigation solutions throughout the root canal system [15].

## Conclusions

Successful endodontic management of complex mandibular molars depends on early recognition and precise diagnosis of aberrant anatomies, such as bifurcated roots, C-shaped canals, and supernumerary roots such as radix entomolaris. Pre-operative limited-field-of-view (FOV) CBCT provides crucial 3D multiplanar imaging to map canal paths, evaluate curvatures, and locate hidden orifices when encountering unusual radiographic outlines or suspected extra roots. Combining CBCT diagnosis with heat-treated NiTi rotary instrumentation and active sonic irrigation improves treatment efficacy, preserves tooth structure, and prevents procedural complications like ledging or perforations. Coupled with modern heat-treated NiTi rotary instrumentation and active sonic irrigation, advanced 3D diagnostic imaging optimizes predictable, long-term therapeutic success in complex endodontic cases.
